# Procalcitonin Levels in ICU Patients with SARS-CoV-2-Associated Viral Sepsis

**DOI:** 10.3390/jcm15093339

**Published:** 2026-04-27

**Authors:** Barbara Adamik, Barbara Dragan, Tomasz Skalec, Piotr Badeński, Anna Kupiec, Małgorzata Grotowska, Lidia Łysenko, Adrianna Lebiedzińska, Agata Chalasiewicz, Agnieszka Matera-Witkiewicz, Adrian Doroszko, Katarzyna Kiliś-Pstrusińska, Michał Pomorski, Marcin Protasiewicz, Janusz Sokołowski, Krzysztof Kaliszewski, Ewa Anita Jankowska, Katarzyna Madziarska

**Affiliations:** 1 Clinical Department of Anesthesiology and Intensive Therapy, Wroclaw Medical University, 50-367 Wrocław, Poland; barbara.dragan@umw.edu.pl (B.D.); tomasz.skalec@umw.edu.pl (T.S.); piotr.badenski@umw.edu.pl (P.B.); anna.kupiec@umw.edu.pl (A.K.); malgorzata.grotowska@umw.edu.pl (M.G.); lidia.lysenko@umw.edu.pl (L.Ł.); 2 Clinical Department of Anesthesiology and Intensive Therapy, University Clinical Hospital, 50-556 Wrocław, Poland; 3 Department of Microbiology, University Clinical Hospital, 50-556 Wrocław, Poland; agata.chalasiewicz@usk.wroc.pl; 4 Screening of Biological Activity Assays and Collection of Biological Material Laboratory, Wroclaw Medical University Biobank, Faculty of Pharmacy, Wroclaw Medical University, 50-367 Wrocław, Poland; agnieszka.matera-witkiewicz@umw.edu.pl; 5 Department of Internal Medicine, Hypertension and Clinical Oncology, Wroclaw Medical University, 50-367 Wroclaw, Poland; adrian.doroszko@gmail.com; 6 Department of Cardiology, 4th Military Hospital, 50-981 Wrocław, Poland; 7 Clinical Department of Paediatric Nephrology, Faculty of Medicine, Wroclaw Medical University, 50-367 Wrocław, Poland; katarzyna.kilis-pstrusinska@umw.edu.pl; 8 Clinical Department of Gynecology and Obstetrics, Wroclaw Medical University, 50-367 Wrocław, Poland; michal.pomorski@umw.edu.pl; 9 Institute of Heart Diseases, Wroclaw Medical University, 50-556 Wroclaw, Poland; mprot@poczta.onet.pl (M.P.); ewa.jankowska@umw.edu.pl (E.A.J.); 10 Clinical Department of Emergency Medicine, Wroclaw Medical University, 50-367 Wrocław, Poland; janusz.sokolowski@umw.edu.pl; 11 Department of General Surgery, University Centre of General and Oncological Surgery, Wroclaw Medical University, 50-367 Wrocław, Poland; krzysztof.kaliszewski@umw.edu.pl; 12 Clinical Department of Diabetology, Hypertension and Internal Diseases, Institute of Internal Diseases, Wroclaw Medical University, 50-367 Wrocław, Poland; katarzyna.madziarska@umw.edu.pl

**Keywords:** procalcitonin, sepsis, viral infection, COVID-19

## Abstract

**Background**: Sepsis has heterogeneous etiologies. Although bacteria are the most common causative agents, viral and yeast forms of sepsis also occur. Procalcitonin (PCT) is widely used to monitor severe bacterial infections and may support the differential diagnosis of infection etiology. **Methods**: We evaluated the diagnostic value of PCT in viral sepsis using PCT levels at ICU admission and PCT kinetics during ICU treatment. **Results**: During the COVID-19 pandemic, 191 adult ICU patients with sepsis and a positive SARS-CoV-2 PCR test at hospital admission were included and classified into two groups according to the presence or absence of bacterial or yeast co-infection. PCT showed a distinct diagnostic pattern influenced by the presence of co-infection. PCT remained low in isolated viral sepsis, whereas elevated concentrations were associated with superimposed bacterial or yeast co-infection and worse clinical outcomes. Overall mortality was significantly lower in patients with isolated viral sepsis compared to those with co-infection (50 vs. 69%, *p* = 0.009). **Conclusions**: In viral sepsis, persistently low PCT concentrations argue against bacterial co-infection, whereas elevated or rising values should prompt increased diagnostic evaluation. Although PCT provides clinically relevant diagnostic information, it must be interpreted cautiously and in conjunction with clinical assessment and microbiological data. PCT should serve as an adjunctive, not a standalone, marker of infection etiology in sepsis.

## 1. Introduction

Procalcitonin (PCT) is an inflammatory marker commonly used to assess and monitor severe bacterial infections. When the immune system is activated, the PCT level increases as multiple tissues begin producing it in response to pathogen-associated signals and circulating inflammatory mediators. In clinical practice, PCT is typically measured once per day, especially in patients with suspected or confirmed bacterial infection. Clinical interpretation emphasizes how values change over time, whether they are falling or remain persistently elevated, rather than relying on a single measurement. In sepsis, serial PCT monitoring reflects the magnitude of the inflammatory response and can offer indirect insight into how well a patient is responding to antibiotic treatment. Sepsis, however, has heterogeneous etiologies: the most common etiologic agents are bacteria, but viral and yeast forms of sepsis also occur. Current SSC guidelines recommend initiating antimicrobial therapy immediately in cases of septic shock or when the likelihood of sepsis is high, regardless of the presumed pathogen [1]. This approach highlights a key challenge: antimicrobial regimens are designed primarily for bacterial infections, raising questions about their appropriateness in viral or yeast sepsis. Distinguishing bacterial from non-bacterial sepsis remains difficult in routine clinical practice [2,3]. Clinicians often struggle to determine which patients truly require antibiotic therapy, as microbiological cultures may take several days to yield results. Although molecular diagnostics can accelerate pathogen identification, they are not universally accessible; consequently, there is continued interest in biomarkers that may help differentiate infection etiologies. Inflammatory markers such as PCT can support clinical assessment, but their diagnostic accuracy is influenced by multiple factors, including the timing of symptom onset, pathogen load and virulence, the nature of pathogen-derived mediators, and patient-specific characteristics of the immune response (for example, whether a fever is present) [4,5,6]. These limitations highlight the need to interpret biomarker data within the broader clinical picture rather than relying on any single parameter in isolation.

Previous studies have examined whether PCT can reliably distinguish bacterial from viral infections, but the findings remain inconsistent. Elevated PCT levels have been linked to worse outcomes in COVID-19, yet it is unclear whether this reflects the inflammatory response to the virus itself or indicates a concurrent bacterial infection that might warrant antibiotic therapy [7]. Williams et al. reported that PCT-guided decision-making in SARS-CoV-2 infection improved diagnostic discrimination and reduced antibiotic use without affecting outcomes [8]. Van Berkel and colleagues found that low PCT levels in ICU patients with confirmed viral infection may help rule out bacterial co-infection [9]. In contrast, Vanhomwegen et al. observed that PCT measured at ICU admission was not a reliable indicator of bacterial co-infection in COVID-19 [10]. A multicenter study by Huang et al. further demonstrated that providing PCT results in the emergency department did not improve diagnostic accuracy or reduce antibiotic use in patients with suspected lower respiratory tract infections, suggesting that clinicians may not consider PCT reliable for distinguishing infection etiologies [11]. In another multicenter study by Galli et al., PCT demonstrated limited ability to identify community-acquired bacterial co-infection in COVID-19 pneumonia, and no significant association was found between PCT levels and 30-day mortality [12].

To address the knowledge gap regarding the role of PCT in distinguishing infection etiology in sepsis, we conducted a study of adult ICU patients during the COVID-19 pandemic. The analysis included both community- and hospital-acquired infections and incorporated microbiological data from a wide range of anatomical sites, including respiratory, bloodstream, urogenital, and intra-abdominal sources, as well as bacterial and yeast co-infections. Importantly, the cohort consisted exclusively of patients meeting sepsis criteria—a population at particularly high risk for secondary infections due to critical illness, immune dysregulation, invasive procedures, and prolonged ICU stay. We evaluated the diagnostic value of PCT at ICU admission and examined its trajectory during ICU treatment.

## 2. Materials and Methods

### 2.1. Patients

This retrospective observational study was conducted in the ICU of Wroclaw University Hospital between August 2020 and May 2021 (spanning 10 months). Consecutive adult patients (≥18 years) admitted with sepsis were screened for eligibility. Inclusion criteria were as follows: (I) age ≥ 18 years; (II) diagnosis of sepsis at the time of ICU admission; (III) COVID-19 infection confirmed by polymerase chain reaction testing of a nasopharyngeal sample, and (IV) availability of plasma PCT measurements initiated within the first 24 h of ICU admission. Exclusion criteria included the absence of daily PCT measurements during ICU treatment. Detailed definitions and procedures for inclusion/exclusion criteria are provided in the paragraphs below. All patients received standard treatment in accordance with Surviving Sepsis Campaign guidelines [1,13,14].

### 2.2. Data Source

All data (demographics, laboratory measurements, comorbidities, and interventions) were retrospectively extracted from the electronic hospital system as part of the COronavirus in the LOwer Silesia (“COLOS”) study. The study protocol was approved by the Institutional Review Board and Bioethics Committee of Wroclaw Medical University, Wroclaw, Poland (No.: KB-444/2021; approved on 21 May 2021). The Bioethics Committee approved the publication of fully anonymized data. The requirement for written informed consent to participate in the study was waived to limit unnecessary contact and transmission of the virus. The research adhered to the principles of the Declaration of Helsinki (1975), as revised in 2013.

### 2.3. Definitions and Laboratory Measurements

Sepsis was defined, according to Sepsis-3 criteria, as suspected or documented infection and acute organ dysfunction with an increase in SOFA score of ≥2 points [15]. The diagnostic time window relative to ICU admission was 24 h. Viral sepsis was defined as sepsis with a confirmed SARS-CoV-2 etiology. To establish viral etiology, SARS-CoV-2 testing was performed in all patients using nasopharyngeal samples collected on the day of ICU admission, within a 24 h window. Viral detection was carried out using the Vitassay qPCR SARS-CoV-2 kit (Vitassay Healthcare S.L.U., Huesca, Spain) and the VIASURE SARS-CoV-2 Real-Time PCR Detection Kit (CerTest Biotec S.L., Zaragoza, Spain).

Microbiological samples were obtained from blood, respiratory specimens, and other anatomical sites, including urine, tracheal aspirates or bronchoalveolar lavage, surgical specimens, and cerebrospinal fluid. Samples were collected as soon as possible, within 24 h of ICU admission, and repeated thereafter based on clinical indications. Bacterial or yeast co-infection was defined as any infectious episode diagnosed by the attending physician, based on clinical presentation, laboratory parameters, and microbiological results. Co-infections identified either at ICU admission or during the 14-day observation period were included. To minimize contamination, strict aseptic techniques, appropriate sampling procedures, and careful interpretation of microbiological findings were ensured.

Organ dysfunction was evaluated using the Sequential Organ Failure Assessment (SOFA) score, which incorporates six organ systems: cardiovascular (mean arterial pressure and vasopressor requirements), respiratory (PaO_2_/FiO_2_ ratio), hepatic (serum bilirubin concentration), renal (serum creatinine concentration), neurological (Glasgow Coma Scale), and coagulation (platelet count). The SOFA score was calculated using the worst values recorded within the first 24 h of ICU admission.

PCT results were retrospectively extracted from the electronic hospital system. The routine protocol for PCT monitoring involved once-daily measurements from serum samples collected during the morning blood draw. For newly admitted ICU patients, a sample for PCT determination was obtained as soon as possible within the first 24 h of ICU admission, transported to the hospital laboratory via the pneumatic tube system, and the result was subsequently recorded in the electronic system. Patients with missing PCT values either at ICU admission or during the 14-day observation period were excluded from the analysis.

### 2.4. Endpoints

Outcomes related to the performance of the PCT measurements were evaluated. The specific objectives were:

1. To assess the diagnostic value of PCT at ICU admission in differentiating viral sepsis with bacterial or yeast co-infection;
2. To compare longitudinal PCT trajectories between patients with and without bacterial or yeast co-infections;
3. To evaluate the distribution of PCT concentrations in patients with viral sepsis alone versus those with additional bacterial or yeast co-infection.

### 2.5. Statistical Analysis

The primary endpoint of the study was the diagnostic performance of PCT at ICU admission for identifying bacterial or yeast co-infection in patients with viral sepsis. Secondary endpoints included the trajectory of PCT levels during the 14-day ICU observation period and the distribution of PCT concentrations in viral sepsis alone versus viral sepsis with co-infection. Continuous variables were summarized as means with standard deviations and their corresponding minimum and maximum values, whereas categorical variables were described using counts and percentages. Group comparisons for continuous data were conducted using the Mann–Whitney U test, while categorical data were evaluated using contingency tables and either the chi-square test or Fisher’s exact test, as appropriate. The predictive performance of PCT levels measured at ICU admission was assessed through receiver operating characteristic (ROC) analysis, with the area under the curve (AUC) and 95% confidence intervals (CI) used to quantify diagnostic accuracy. AUC values were interpreted as follows: 0.5 indicating no discrimination, 0.6–0.7 poor, 0.7–0.8 acceptable, 0.8–0.9 very good, and 0.9–1.0 excellent. The optimal threshold for predicting co-infection in patients with viral sepsis was determined using Youden’s index. Missing data were handled using complete-case analysis. Patients with missing PCT measurements at ICU admission or with incomplete PCT data during the 14-day observation period were excluded from the study, as prespecified in the protocol. A two-sided *p*-value below 0.05 was considered indicative of statistical significance. All statistical procedures were carried out in Statistica version 13.3 (TIBCO Software Inc., Palo Alto, CA, USA) under a license held by Wroclaw Medical University.

## 3. Results

### 3.1. Baseline Characteristics

Between February 2020 and June 2021, 2183 adult patients with a positive SARS-CoV-2 PCR test at hospital admission were treated. Of these, 216 required ICU care: 191 met the study’s inclusion criteria, while 25 were excluded due to missing PCT data. At hospital admission, PCT concentrations differed significantly between patients who were later admitted to the ICU and those who were not (1.8 ± 7.0 ng/mL, range 0.0–72.6 ng/mL, vs. 1.0 ± 5.3 ng/mL, range 0.0–66.5 ng/mL; *p* < 0.001).

In the ICU cohort (N = 191), most patients (62%) were transferred from the emergency department or another hospital ward within 24 h, while 38% arrived from wards where they had already been treated for 2–36 days. In all patients, viral presence was reconfirmed upon ICU entry. All patients presented with severe respiratory failure secondary to COVID-19 pneumonia, and 89% met the criteria for acute respiratory distress syndrome. Sepsis of viral etiology, with SARS-CoV-2 as the presumed pathogen, was diagnosed in all cases according to Sepsis 3 criteria, and septic shock occurred in 70% of patients. In some patients, bacterial or yeast pathogens were additionally identified either at ICU admission or during the 14-day observation period. Based on the presence of bacterial or yeast co-infection, patients were categorized into two groups:

- Group 1: Viral infection only (N = 64);
- Group 2: Viral infection with bacterial or yeast co-infection (N = 127).

During ICU treatment, PCT levels were measured daily. Detailed comparisons between groups are presented in subsequent sections. Patients in Group 2 showed more severe illness at ICU admission, as reflected by higher SOFA scores. All patients in both groups required respiratory support, with the majority receiving mechanical ventilation. All patients, both those with viral infection alone and those with viral infection accompanied by bacterial or yeast co-infection, received antibiotic treatment in the ICU in accordance with SSC guidelines. Antibiotic therapy was initiated within the first 48 h of hospital admission (ICU or other ward) in 75% of patients without co-infection (N = 48), compared with 87% of those with ICU-confirmed co-infection (N = 110) (*p* = 0.045). Overall, 26% of patients (N = 49) had an elapsed time of >48 h between the initiation of antimicrobial therapy and ICU admission (21 patients without co-infection and 28 with co-infection, *p* = 0.107). Overall mortality was 63% and was significantly lower in Group 1 than in Group 2 (50% vs. 69%, *p* = 0.009). Demographic and clinical characteristics of both groups are summarized in Table 1.

### 3.2. Microbiology/Co-Infection Patterns

To characterize the microbiological profile of co-infections in viral sepsis, we examined the timing of co-infection onset, the spectrum of identified pathogens, and their sites of isolation. Among the 127 patients in Group 2 (viral sepsis with additional bacterial or yeast co-infection), 43% (N = 54) had a confirmed co-infection based on samples collected at ICU admission, while 72% of patients (N = 92) developed co-infection during their ICU stay.

Co-infections present at ICU admission (N = 54): In this subgroup, Gram-positive bacteria were the most frequently identified pathogens (56%), followed by Gram-negative bacteria (50%) and atypical organisms such as Mycoplasma pneumoniae and Chlamydia pneumoniae (5%). Yeast infections were not detected at this stage. Most patients had a single bacterial pathogen (87%), whereas polymicrobial infections occurred in 13%. The respiratory tract was the predominant site of pathogen isolation (50%), followed by bloodstream infections (41%), the urogenital tract (15%), and the abdominal cavity (7%).

Co-infections acquired during ICU stay (N = 92): In this subgroup, Gram-negative pathogens predominated (84%). Gram-positive organisms were identified in 38% of samples, and yeasts in 3%. Single pathogen infections were observed in 74% of cases, while 26% had polymicrobial infections. The respiratory tract remained the most common site of pathogen isolation (77%), followed by blood (39%), the urogenital tract (8%), and the abdominal cavity (2%). A detailed list of all isolated microorganisms, including their number and percentage, both at ICU admission and those acquired during the ICU stay in patients with co-infection, has been added as Supplementary Table S1.

### 3.3. PCT at Admission

**Figure 1:**
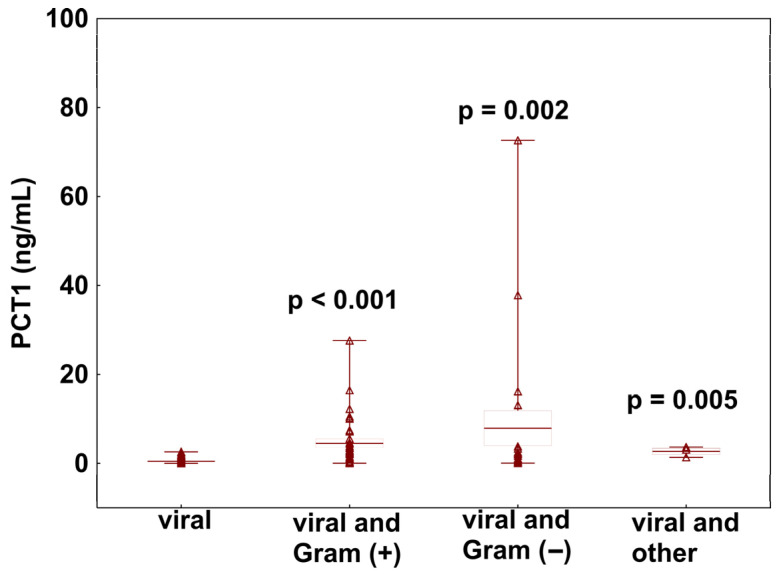
PCT levels measured at ICU admission in patients with viral sepsis, with or without bacterial co-infection, further stratified by bacterial type. *p*-values reflect subgroup comparisons against the viral-only group. “Other” includes Mycoplasma pneumoniae and Chlamydia pneumoniae.

To assess the diagnostic value of PCT in differentiating viral sepsis alone from viral sepsis with bacterial co-infection at the time of ICU admission, PCT concentrations measured on day 1 were evaluated. Patients without co-infection had markedly lower PCT levels compared with those with co-infection (0.46 vs. 5.66 ng/mL, *p* < 0.001). Among patients with co-infection, stratified analysis revealed mean PCT concentrations of 4.49 ± 5.86 ng/mL (range: 0.03–27.59) in Gram-positive infections, 7.91 ± 17.66 ng/mL (range: 0.06–72.61) in Gram-negative infections, and 2.69 ± 1.19 ng/mL (range: 1.35–3.66) in other bacterial co-infections (including Mycoplasma pneumoniae and Chlamydia pneumoniae). Post hoc testing using the Kruskal–Wallis ANOVA confirmed statistically significant differences in PCT levels across these subgroups (Figure 1).

### 3.4. PCT Kinetics

**Figure 2:**
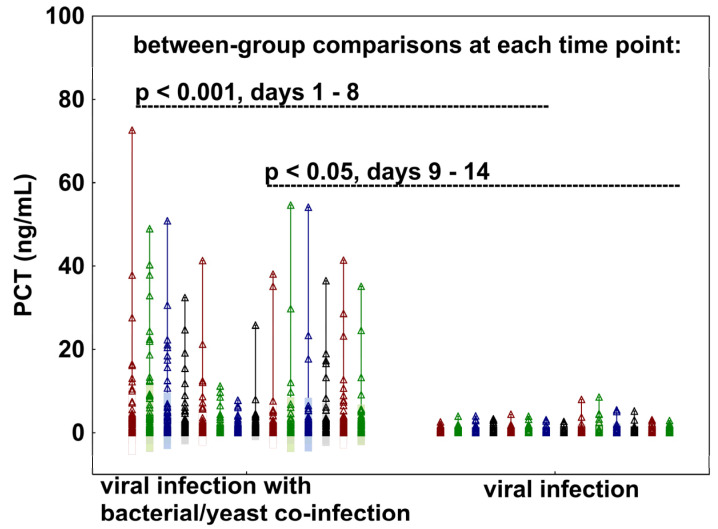
Changes in PCT levels over a 14-day observation period in patients with viral sepsis with (**left panel**) and without (**right panel**) bacterial or yeast co-infection. *p*-values indicate between-group comparisons at each time point, demonstrating statistically significant differences throughout the study period.

To better characterize the impact of co-infection on PCT levels in viral sepsis, we compared longitudinal PCT trajectories between patients with and without bacterial or yeast co-infections. Across the entire 14-day observation period, PCT levels remained significantly lower in patients with viral sepsis than in those with viral sepsis complicated by co-infection. Between-group comparisons showed *p*-values <0.001 on days 1–8 and <0.05 on days 9–14, indicating consistent statistical significance (Figure 2). The two groups demonstrated clearly divergent PCT trajectories. Patients with viral sepsis and bacterial or yeast co-infection exhibited elevated PCT levels at admission, followed by a decline during treatment and a subsequent rise later in the course, likely reflecting the development of secondary bacterial or yeast co-infections. In contrast, patients with viral sepsis without co-infection maintained persistently low PCT concentrations throughout the 14-day period.

### 3.5. Distribution of PCT Levels

To provide an overall view of PCT concentrations in both groups, we examined the distribution of all PCT measurements collected during the 14-day observation period. Most PCT values in both groups remained below 1 ng/mL: 86% of all measurements in patients with isolated viral infection and 70% in those with viral infection plus bacterial or yeast co-infection. PCT concentrations above 1 ng/mL were less common, occurring in 14% of measurements in Group 1 and 30% in Group 2. Occasional high PCT values in patients with viral sepsis only may reflect an intensified individual inflammatory response; however, undetected bacterial or yeast co-infection cannot be entirely excluded. The full distribution of PCT values recorded during the 14-day period for both groups is presented in Table 2.

### 3.6. Diagnostic Utility of PCT Measured on ICU Admission

**Figure 3:**
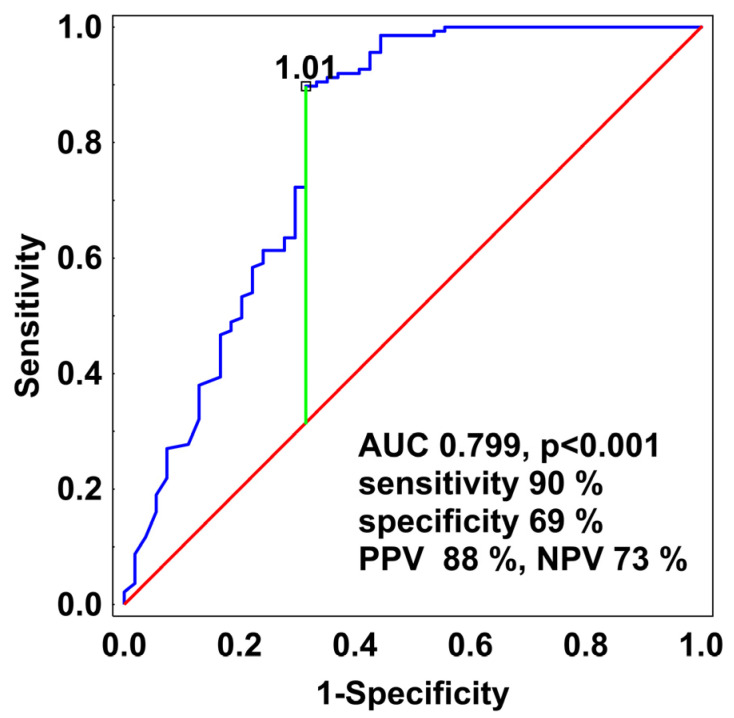
ROC curve illustrating the discriminative performance of PCT for excluding bacterial co-infection at ICU admission. The optimal cut-off value, determined using Youden’s index, was 1.01 ng/mL.

To assess the diagnostic value of PCT at ICU admission for distinguishing viral sepsis with bacterial or yeast co-infection, we calculated the area under the ROC curve (AUC) and determined the optimal cut-off value. ROC analysis demonstrated acceptable discriminative performance (AUC = 0.799, 95% CI 0.717–0.881, *p* < 0.001), indicating that PCT can reliably differentiate between patients with and without co-infection at ICU admission. Using Youden’s index, the optimal cut-off value was determined to be 1.01 ng/mL (Figure 3). At this threshold, sensitivity was 90% (95% CI: 84–94%), specificity 69% (95% CI: 54–81%), accuracy 84% (95% CI: 78–89%), positive predictive value (PPV) 88% (95% CI: 81–93%), and negative predictive value (NPV) 73% (95% CI: 58–83%). However, interpretation of this cut-off requires caution. Thirty percent of patients with viral infection and bacterial co-infection had PCT levels below 1.01 ng/mL at admission, underscoring that antibiotic therapy should not be withheld solely on the basis of PCT. Conversely, 10% of patients without confirmed co-infection had PCT values above the cut-off, raising the risk of unnecessary antibiotic exposure. Overall, PCT shows good, but not definitive, discriminative ability for identifying bacterial co-infection in viral sepsis, supporting its use as an adjunctive marker rather than a standalone criterion for guiding antibiotic therapy.

## 4. Discussion

In this cohort of critically ill patients with viral sepsis, PCT displayed distinct diagnostic patterns that were strongly influenced by the presence of bacterial or yeast co-infection. The molecular basis for the diagnostic utility of circulating PCT in distinguishing bacterial from viral sepsis is outlined in the following paragraph. Our findings indicate that while PCT remains largely suppressed in isolated viral sepsis, its elevation is associated with superimposed bacterial or yeast infection and with poorer clinical outcomes. At the same time, its incomplete sensitivity highlights the need for careful interpretation when incorporating PCT into clinical decision-making to complement, not replace, clinical judgment.

PCT has been extensively investigated as a potential marker for differentiating the etiology of infection, given its characteristic elevation in systemic bacterial infections. The diagnostic utility of circulating PCT has a well-defined molecular basis. PCT is encoded by the CALC I gene on chromosome 11. Although under physiological conditions CALC I expression is largely restricted to thyroid C cells, sepsis induces a marked upregulation of CALC I transcription across multiple extrathyroidal tissues, resulting in substantial increases in circulating PCT concentrations [16]. Importantly, PCT production during infection is independent of thyroid tissue and instead reflects a systemic, inflammation-driven response. During bacterial infections, microbial components such as lipopolysaccharide, together with host-derived proinflammatory cytokines including interleukin-1β and tumor necrosis factor-α, induce widespread CALC I gene expression and subsequent PCT release [17]. In contrast, interferons, particularly those involved in early antiviral defense, exert a potent inhibitory effect on CALC I gene transcription [17,18]. This antagonistic interaction between cytokine-mediated stimulation and interferon-mediated suppression provides a mechanistic explanation for the clinically observed pattern in which viral infections are typically associated with only modest increases in circulating PCT, whereas bacterial infections lead to marked elevations [19].

### 4.1. PCT in Viral Sepsis and the Impact of Co-Infection

In the present cohort, patients with confirmed viral sepsis and without documented bacterial or yeast co-infection exhibited persistently low PCT concentrations throughout the 2-week ICU observation period. This finding supports the concept that virus-driven systemic inflammation does not consistently induce PCT release. In contrast, patients with viral sepsis and confirmed bacterial or yeast co-infection showed significantly higher PCT levels with clearly distinct longitudinal patterns, characterized by early elevation, subsequent decline following antimicrobial therapy, and, in some cases, a secondary increase. The latter likely reflects the development of secondary nosocomial infections during the ICU stay. These findings are concordant with prior evidence. A meta-analysis by Lippi et al. demonstrated that PCT concentrations remain low in patients with uncomplicated SARS-CoV-2 infection, whereas marked increases are associated with bacterial co-infection in patients who develop severe disease [20]. Importantly, suppression of PCT induction does not appear to be unique to SARS-CoV-2. In patients with community-acquired pneumonia primarily caused by influenza A (H1N1), Rodriguez et al. reported lower PCT levels at ICU admission compared with those observed in cases of bacterial co-infection [19]. In contrast, Kamat et al. reported that, in the broader context of community-acquired pneumonia of mixed etiologies, a single PCT measurement is unlikely to reliably distinguish bacterial from viral infection [21]. Taken together, these data suggest that isolated PCT measurements, particularly early in the course of infection, have limited diagnostic utility, as the magnitude of PCT elevation is influenced by the timing of symptom onset [5]. In contrast, daily PCT measurements may offer additional clinical insight into disease evolution, facilitate the detection of secondary bacterial co-infection, and help identify patients at risk of clinical deterioration.

### 4.2. Microbiological Patterns and PCT Heterogeneity

The pattern of co-infections changed over the course of the ICU stay. Gram-positive pathogens predominated at ICU admission, whereas Gram-negative organisms were more frequently isolated during ICU treatment, consistent with the previously reported shift from early acquired to later hospital-acquired infections [19,22,23]. Gram-negative infections were associated with higher PCT concentrations than Gram-positive or atypical pathogens. These findings align with experimental and clinical data indicating stronger PCT induction in response to lipopolysaccharide-mediated inflammation [24,25,26]. Because Gram-negative bacteria contain lipopolysaccharide, a potent activator of innate immune signaling predominantly through the TLR4/NF-κB pathway, they tend to elicit higher PCT levels, whereas Gram-positive infections generally produce lower concentrations. Intermittent PCT elevations in patients without microbiologically documented co-infection warrant cautious interpretation. Such fluctuations may reflect individual variability in the inflammatory response or limitations of routine microbiological diagnostics, potentially resulting in undetected infections. Since microbiological confirmation of infection is achieved in only about 70% of sepsis cases, negative cultures do not reliably exclude bacterial involvement, particularly in critically ill patients receiving early empirical antimicrobial therapy [27,28].

### 4.3. Diagnostic Utility of PCT at ICU Admission

The ability of PCT measured at ICU admission to distinguish the etiology of sepsis remains uncertain. Although PCT may contribute complementary information, its diagnostic value is limited by substantial overlap in concentrations observed in bacterial, viral, and mixed infections. In our cohort, admission PCT demonstrated acceptable discriminative performance for identifying bacterial co-infection, with an AUC of 0.79 and an optimal cut-off of 1.01 ng/mL. At this threshold, sensitivity was 90% and specificity 69%, with a positive predictive value of 88%. However, nearly one-third of patients with confirmed co-infection had PCT values below this cut-off, indicating a clinically relevant false-negative rate. Conversely, elevated PCT levels in some patients without documented co-infection raise concerns about false positive results and potential antibiotic overuse. Prior studies have reported similar limitations. Cuquemelle et al. found that although PCT values were generally <1.0 ng/mL in isolated viral pneumonia, occasional high values occurred, reducing specificity [29]. Using a cut-off of 0.8 µg/L, PCT achieved high sensitivity (91%) but only moderate specificity (68%), with an AUC of 0.90. In influenza A (H1N1) pneumonia, a 1.0 ng/mL cut-off yielded a sensitivity of 66%, a specificity of 64%, and a high negative predictive value of 88% (AUC 0.71) for detecting bacterial co-infection [19]. Van Berkel et al. reported low PCT levels in ICU patients with COVID-19, with significant increases in those who developed secondary bacterial co-infection; at a 1.0 ng/mL cut-off, sensitivity was 48% and specificity 96% (AUC 0.80) [9]. In the study by Galli et al., PCT demonstrated limited ability to detect community-acquired bacterial co-infection in COVID-19 pneumonia [12]. Although a threshold of ≥0.12 ng/mL yielded a sensitivity of 81% and an NPV of 97%, the low AUC reflected poor overall performance. The authors further underscored that a single PCT measurement is insufficient for reliably diagnosing bacterial co-infection. Taken together, these findings indicate that while PCT may aid in the etiological assessment of sepsis, its diagnostic performance varies substantially across clinical settings and patient populations. PCT should therefore be interpreted as an adjunctive marker and integrated with clinical evaluation rather than as a standalone determinant of infection etiology in sepsis.

### 4.4. Bacterial Co-Infection in Critically Ill COVID-19 Patients

Bacterial co-infections in COVID-19 vary widely, and their true burden remains uncertain due to substantial differences in study design. A meta-analysis of SARS-CoV-2 infections across community and hospital settings estimated a prevalence of about 8.6%, but the authors noted that only a few studies included microbiological confirmation and that heterogeneity limited the reliability of pooled estimates [30]. Higher rates have been described in critically ill populations. In patients who died from COVID-19 pneumonia, bacterial or yeast pulmonary co-infection was identified in 38.5% of cases [31], and a pilot study of severe COVID-19 reported co-infection rates up to 80% [32]. Similarly, in the multicenter CIBERESUCICOVID study of ICU-admitted patients with SARS-CoV-2 pneumonia, Torres et al. reported hospital-acquired, microbiologically confirmed bacterial co-infections in 20% of respiratory tract samples and 28% of blood samples, although other anatomical sites and yeast pathogens were not assessed [33]. In contrast, a subanalysis of the same cohort by Galli et al., focusing only on community-acquired co-infections at admission, found a prevalence of just 3%, illustrating how timing, definitions, and sampling strategies strongly influence reported rates [12]. In the present study, we evaluated co-infections in patients with SARS-CoV-2-related sepsis without restricting analyses to community- or hospital-acquired infections. This broader approach, together with the inclusion of critically ill patients, likely reflects a population at high risk for secondary infections due to immune dysregulation, invasive procedures, and prolonged ICU stay, which may explain the higher co-infection rates. Importantly, wide variability in co-infection rates is not unique to COVID-19. A systematic review of influenza-associated bacterial co-infection reported estimates ranging from 2% to 65%, underscoring the influence of methodological differences and patient selection [34]. During the COVID-19 pandemic, several factors likely contributed to inconsistent reporting of co-infection rates. Microbiological testing was frequently limited due to the overwhelming clinical workload and efforts to minimize healthcare worker exposure. Diagnostic procedures such as bronchoscopy or induced sputum collection were often restricted because of aerosol-generating risk. Early empiric antimicrobial therapy may have further reduced culture yield, making co-infections harder to detect. Overall, these findings highlight the need for systematic microbiological diagnostics in patients with sepsis of viral origin to accurately determine co-infection prevalence and guide appropriate antimicrobial use.

### 4.5. Limitations of the Study

This study has several limitations. First, its single-center, retrospective design may limit its generalizability. Nevertheless, given the limited evidence on ICU patients with viral-origin sepsis, these findings still add valuable insight into PCT kinetics in this setting. Second, patients were admitted during different waves of the COVID-19 pandemic, and these periods were not analyzed separately. Differences in circulating viral variants, patient characteristics, and treatment practices may therefore have introduced heterogeneity. Third, bacterial or yeast co-infection was restricted to microbiologically confirmed cases. While this approach increases diagnostic specificity, it may have led to missed cases of microbiologically unconfirmed co-infection. Finally, the results apply specifically to sepsis caused by SARS-CoV-2 and may not be directly transferable to sepsis from other viral pathogens.

## 5. Conclusions

In critically ill patients with viral sepsis, persistently low PCT concentrations argue against the presence of bacterial co-infection, whereas elevated or rising values should prompt increased diagnostic evaluation. Although PCT demonstrates clinically relevant diagnostic utility, its limitations require cautious interpretation and integration into a broader clinical assessment. PCT is best used as an adjunctive biomarker, interpreted alongside clinical evaluation and microbiological data, rather than as a standalone indicator of infection etiology in sepsis.

## Figures and Tables

**Table 1:** Demographic and clinical characteristics of patients.

Parameter	Group 1	Group 2	*p*
	N = 64	N = 127	
**Age, (years)**	61 ± 14 (26–90)	60 ± 14 (23–87)	0.486
**Male sex, N (%)**	41 (64)	87 (69)	0.537
**SOFA, (points)**	8 ± 3 (2–18)	9 ± 3 (4–21)	0.033
**ARDS, N (%)**	54 (84)	117 (92)	0.014
**Pneumonia, N (%)**	64 (100)	127 (100)	-
**Sepsis, N (%)**	64 (100)	127 (100)	-
**Septic shock, N (%)**	38 (59)	95 (75)	0.028
**Comorbidities, N (%):**			
Hypertension	37 (58)	74 (58)	0.952
Ischemic heart disease	8 (13)	19 (15)	0.644
Pulmonary disease	6 (9)	10 (8)	0.723
Diabetes	21 (33)	47 (37)	0.567
Chronic kidney disease	5 (8)	14 (11)	0.484
Cerebrovascular disease	3 (5)	7 (6)	0.554
Active cancer	4 (6)	10 (8)	0.466
Smoking	3 (5)	9 (7)	0.790
Obesity	17 (27)	45 (35)	0.234
**Treatment, N (%):**			
Antimicrobials	64 (100)	127 (100)	-
Corticosteroids	56 (88)	101 (80)	0.173
Respiratory support:			0.034
Noninvasive	7 (11)	10 (8)	
Mechanical ventilation	57 (89)	117 (92)	
Vasopressors	31 (48)	79 (62)	0.244
CRRT	5 (8)	7 (6)	0.311
**Length of stay before ICU, (days)**	5 ± 7 (1–31)	4 ± 7 (1–36)	0.091
**Length of stay in ICU, (days)**	9 ± 6 (3–30)	18 ± 17 (3–128)	<0.001
**Mortality, N (%)**	32 (50)	88 (69)	0.009

SOFA, Sequential Organ Failure Assessment; ARDS, acute respiratory distress syndrome; CRRT, continuous renal replacement therapy; ICU, intensive care unit; LOS, length of stay. Continuous variables are presented as means ± standard deviation (minimum–maximum), and categorical variables are summarized as counts and fractions. The *p*-value represents differences between the groups.

**Table 2:** Distribution of PCT values measured in both groups over the 14-day study period.

PCT Values [ng/mL]	Group 1	Group 2
<1.0	86%	70%
1.0≤ and <2.0	7%	12%
2≤ and <10.0	7%	14%
≥10.0	0	4%

## Data Availability

The data presented in the study are available on request from the 496 corresponding author. The data have not been made publicly available because they contain information that could compromise the privacy of the study participants.

## Supplementary Materials

The following supporting information can be downloaded at https://www.mdpi.com/article/10.3390/jcm15093339/s1, Table S1. Microorganisms isolated at ICU admission and during the ICU stay in patients with viral sepsis and bacterial/yeast co-infection.

## Abbreviations

The following abbreviations are used in this manuscript:

PCT	Procalcitonin
ICU	Intensive care unit
SSC	Surviving Sepsis Campaign
COVID-19	COronaVIrus Disease of 2019
SARS-CoV-2	Severe acute respiratory syndrome coronavirus 2
SOFA	Sequential Organ Failure Assessment
PaO_2_/FiO_2_	Partial pressure of oxygen/fraction of inspired oxygen
AUC	Area under the curve
ROC	Receiver operating characteristic
CRRT	Continuous renal replacement therapy
LOS	length of stay
PPV	Positive predictive value
NPV	Negative predictive value
