# Supplementary material for: Procalcitonin Levels in ICU Patients with SARS-CoV-2-Associated Viral Sepsis
Source: J Clin Med. 2026 Apr 27;15(9):3339. doi: 10.3390/jcm15093339 (PMC13163896; doi:10.3390/jcm15093339)
Supplement: Supplementary file 1 [file jcm-15-03339-s001.zip › jcm-4233088-supplementary.pdf]

Supplementary Material

Table S1. Microorganisms isolated at ICU admission and during the ICU stay in patients with viral sepsis and bacterial/yeast co-infection.

	Microorganisms isolated at ICU admission, N (%)	Microorganisms isolated during ICU stay, N (%)
Enterococcus faecium	13 (24)	12 (13)
Coagulase-negative staphylococci	9 (17)	11 (12)
Enterococcus faecalis	4 (7)	4 (4)
Staphylococcus aureus	4 (7)	7 (8)
Bacillus sp.	0	1 (1)
Acinetobacter baumannii	10 (18)	39 (42)
Klebsiella pneumoniae	7 (13)	23 (25)
Escherichia coli	5 (9)	2 (2)
Serratia marcescens	2 (4)	1 (1)
Stenotrophomonas maltophilia	1 (2)	5 (5)
Proteus mirabilis	1 (2)	2 (2)
Pseudomonas aeruginosa	0	4 (4)
Enterobacter cloacae	1 (2)	1 (1)
Citrobacter koseri	0	1 (1)
Mycoplasma pneumoniae	2 (4)	0
Chlamydia pneumoniae	1 (2)	0
Candida sp.	0	3 (3)
